# Power balance and efficiency of metasurface antennas

**DOI:** 10.1038/s41598-020-74674-w

**Published:** 2020-10-15

**Authors:** Modeste Bodehou, David González-Ovejero, Christophe Craeye, Stefano Maci, Isabelle Huynen, Enrica Martini

**Affiliations:** 1 ICTEAM Institute, Université catholique de Louvain, Place du Levant 3, 1348 Louvain-la-Neuve, Belgium; 2 Univ Rennes, CNRS, Institut d’Electronique et de Télécommunications de Rennes (IETR), UMR 6164, 35000 Rennes, France; 3 Department of Information Engineering and Mathematics, University of Siena, 53100 Siena, Italy

**Keywords:** Electrical and electronic engineering, Applied physics

## Abstract

This paper presents two methods for the efficient evaluation of the power balance in circular metasurface (MTS) antennas implementing arbitrary modulated surface impedances on a grounded dielectric slab. Both methods assume the surface current in the homogenized MTS to be known. The first technique relies on the surface current expansion with Fourier-Bessel basis functions (FBBF) and proceeds by integration of the Poynting vector on a closed surface. The second method is based on the evaluation of the residue of the electric field spectrum at the surface-wave (SW) pole, and is demonstrated by using a current expansion in Gaussian ring basis functions (GRBF). The surface current expansions can be directly obtained either by analyzing the antenna with a Method of Moments (MoM) tool for homogenized MTSs based on FBBF or GRBF, or derived by a projection process. From there, the power contributions, namely the total power delivered by the feed, the radiated power, the SW power, and the Ohmic power losses in the dielectric are computed. Several efficiency metrics are presented and discussed: tapering efficiency, conversion efficiency, loss factor, and diffraction factor. Since the MTS apertures at hand are leaky-wave (LW) antennas, the designer must find a compromise between the aperture efficiency and the conversion efficiency. This requires accurate and fast computational techniques for the efficiency. The present paper demonstrates for the first time that the efficiency of MTS antenna devices can be accurately evaluated in a few minutes. The compromise that should be made during the design process between the tapering efficiency and the conversion efficiency is highlighted. The impact on the efficiency of isotropic versus anisotropic MTS, uniform versus non-uniform modulation index, is analyzed. An excellent agreement is obtained between both approaches, commercial software, and experimental data.

## Introduction

Among different types of metasurfaces (MTSs)^1–6^, modulated MTS antennas are an emerging class of radiating apertures that exploit the modulation of a surface impedance in such a way that an excited cylindrical surface-wave (SW) is transformed into leaky-waves (LW)^7–16^. The SW is in general launched by a transverse magnetic (TM) feed, such as a simple monopole placed at the center of the MTS. The theoretical premises of this technology go back to the work of Oliner and Hessel^17^, in which a rigorous treatment of SW propagation over sinusoidally modulated impenetrable reactive boundary conditions was provided. Over the past decade, this work has inspired many researchers in the antenna community and has led to practical devices with high performance^11–14,17,18^. For example, the introduction of anisotropy in the surface reactance in combination with a variation of the modulation depth and the local period have been used to synthesize a wide range of aperture fields with excellent beam polarization purity and tapering efficiency^18–20^. In addition, very general reactance modulation (i.e. not necessarily locally sinusoidal) based on the solution of the electric field integral equation (EFIE) has been proposed recently to improve the beam shaping capability^21^ as well as multibeam operation^22,23^. After the synthesis of the required impedance modulation, the surface impedance is usually implemented using sub-wavelength patches, printed on a grounded dielectric slab^24^. A progressive change of the parameters describing the patches (orientation, size, etc) allows one to mimic the required impedance on a Cartesian regular lattice. This implementation can be carried out quite accurately as can be seen from the comparison of the surface reactance and the physical MTS structure simulations^25,26^. This means that the antenna performance can be accurately predicted at the homogenized (surface impedance) level, which is much more computationally efficient, as demonstrated by the EFIE techniques in^26,27^. Those techniques provide an excellent computational performance through the discretization of the EFIE involving the grounded substrate Green’s function^28^ kernel, and using appropriate entire-domain basis functions. However, the usage of the substrate Green’s function assumes the substrate to be infinite, and therefore does not properly deal with possible reflection/diffraction effects that stem from the substrate truncation, when the SW is not sufficiently attenuated before reaching the substrate boundary. Diffraction and reflection occurring at the substrate’s edges may produce sidelobes which are not predicted by the simulation models based on the infinite substrate Green’s function when a significant power level reaches the MTS rim. Ohmic power losses may also be non negligible at high frequencies and should be incorporated in the analysis. It is therefore relevant to have a clear understanding, during the design process, of the power balance in MTS antennas.

In^29^, the efficiency of MTS antennas has been treated with the flat optics (FO) formalism^30^. However, this approach, although providing closed-form formulas, is limited to locally sinusoidal reactance modulation. The present paper aims at providing two efficient semi-analytical methods for the rapid and accurate evaluation of the efficiency of MTS antennas. The proposed methods are not necessarily limited to locally sinusoidal modulations. That means, the methods can handle arbitrary surface impedance profiles i.e. based on any synthesis method^7–23^. The derivation will rely on the mathematical formalisms introduced in^26,27^, where the MTS is modeled as a sheet (penetrable) impedance on top of the substrate. This modeling allows one to finely take into account the substrate dispersion, and therefore provides more accurate results in comparison with the opaque (impenetrable) impedance modeling^25^. In^27^, the current distribution has been expanded into Fourier-bessel basis functions (FBBFs), while^26^ uses Gaussian ring basis functions (GRBFs). The choice of those bases in the present paper, besides the fact they provide a fast MoM solution, is justified by their complementary properties. Along the radial coordinate, while FBBFs exhibit selectivity in the spectral domain, GRBFs are selective in space domain. Therefore, they are complementary in describing the fields features. In this paper, FBBFs are used to compute the total power delivered by the feed on the basis of the Poynting theorem. GRBFs current are exploited to compute the SW power flowing beyond the MTS using the residue theorem. Therefore, developing both approaches provides a good cross-validation methodology.

**Figure 1:**
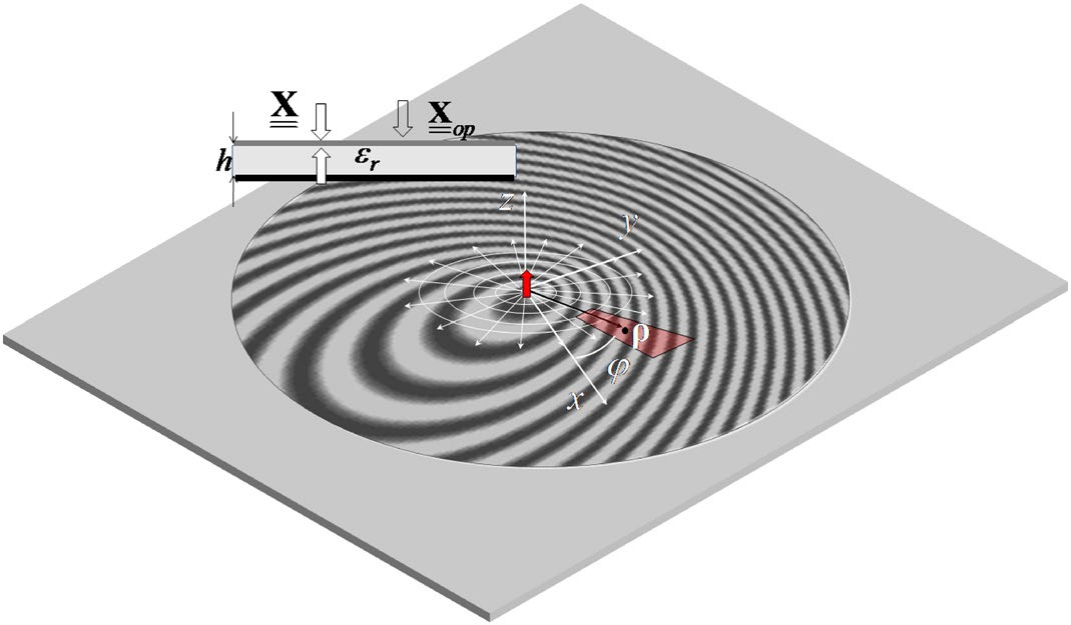
Geometry for the metasurface antenna.

## Results

### Power contributions

#### Illustration and methodology

Let us consider a circular-domain MTS printed on a grounded dielectric slab, as depicted in Fig. 1. The MTS is assumed in this paper to be fed at its center ($\rho =0$) with a vertical elementary dipole. Nevertheless, the methods presented below are also applicable for an arbitrary excitation, assuming the excitation fields are a priori known.

**Figure 2:**
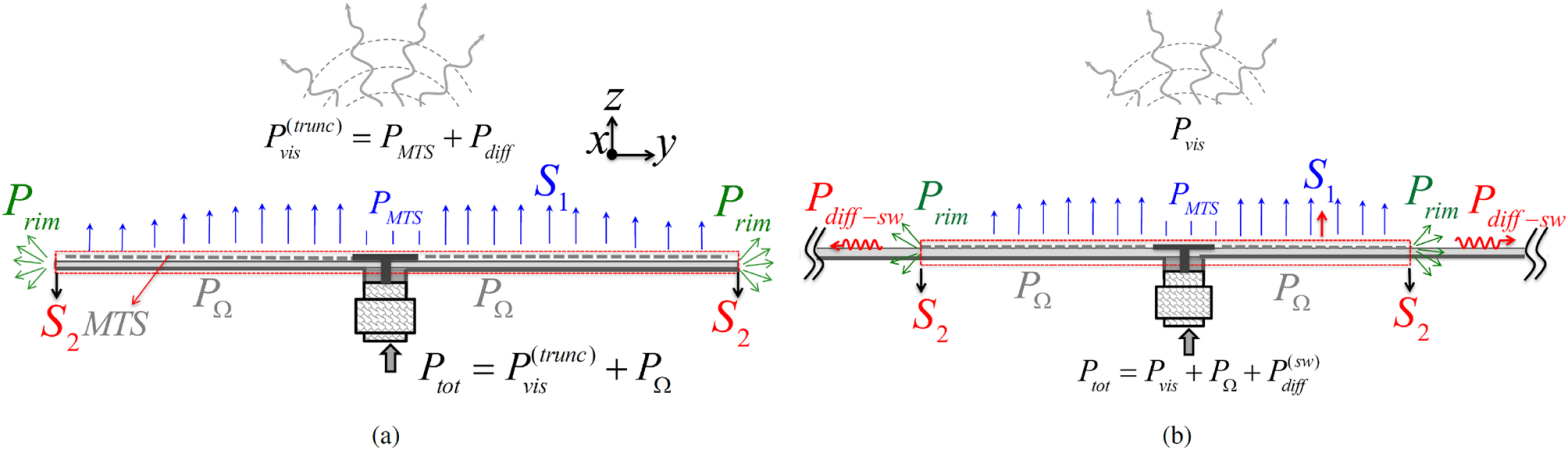
Illustration of the power contributions. (**a**) Finite MTS over a finite grounded substrate. (**b**) Finite MTS over an infinite grounded substrate. The MTS is fed with a coax terminated by a circular patch.

Two different power descriptions are adopted depending on whether the substrate is truncated (Fig. 2a) or infinite (Fig. 2b), the latter occurring in the full wave analysis with the Green’s function of the infinite slab. Although the schematization is approximate, it helps to understand the power balance. When the slab is truncated, the total power $P_{tot}$ delivered by the feed can be calculated by integrating the flux of the real part of the Poynting vector through the dashed red surface in Fig. 2a. This can be subdivided in the integration over the aperture $S_{1}$ and the one over the lateral rim $S_{2}$. We denote these two contributions as $P_{MTS}$ and $P_{rim}$, respectively. The summation of these two contributions gives the radiated power $P_{vis}^{trunc}$, namely the power associated with the visible contribution of the spectrum (far-field). We note that, although $P_{rim}$ is the main contribution associated with the diffracted field, it is not the only one, being also present the asymptotic end point contribution from $S_{1}$. If losses are present, the total power delivered by the feed can be written as $P_{tot}=P_{vis}^{trunc}+P_{\Omega }$, where $P_{\Omega }$ is the power lost in the dielectric and in the metal.

When the grounded substrate is infinite, it is convenient to use a surface of integration like the one in Fig. 2b, which cuts the dielectric substrate. Now, one more term occurs in the power balance, namely the power that is transported by the SW excited at the rim of the printed MTS. We call this term $P_{diff}^{sw}$; it is not visible in the far-field and therefore, the power balance is written as

1 $$\begin{aligned} P_{tot}=P_{vis}+P_{\Omega }+P_{diff}^{sw}~. \end{aligned}$$

Since $P_{tot}$ and $P_{\Omega }$ are almost the same in the two cases, we may say that $P_{vis}^{trunc} \approx P_{vis}+P_{diff}^{sw}$. The contribution $P_{diff}^{sw}$ is in general undesired and leads to a limited conversion efficiency of the antenna. It is worth noting that we do not distinguish here the visible (radiated) field from the surface, namely converted from the SW in LW, and the one radiated directly from the feed. In other terms, we are not interested here in estimating the “feed efficiency”, which is treated in^29^.

The first goal of the present paper consists in evaluating the power contributions, i.e., estimating $P_{vis}$, $P_{diff}^{sw}$, $P_{tot}$, and $P_{\Omega }$ for a given MTS antenna and excitation. The first method, based on FBBFs, computes $P_{vis}$, $P_{\Omega }$, $P_{tot}$, then deduces $P_{diff}^{sw}=P_{tot}-P_{vis}-P_{\Omega }$. The second method, based on GRBFs, computes $P_{vis}$, $P_{diff}^{sw}$, and $P_{\Omega }$, and then derives $P_{tot}=P_{vis}+P_{\Omega }+P_{diff}^{sw}$. This means that the two methods are not only different from the used set of basis functions, but also in term of the physical power contributions that are directly estimated.

In the following, it is assumed that each component of the surface current ${\mathbf{J}}$ in a Cartesian unit vector system ($\hat{\varvec{x}}, \hat{\varvec{y}}$) is known in a Fourier-Bessel basis^27^ or Gaussian ring basis^26^

2 $$\begin{aligned} {\mathbf{J}} (\rho ,\phi )= \sum _{n=-N}^{N} \sum _{m=1}^{M} i_{mn}^{x} R_{m,n}^{x}\left( \rho ,\phi \right) \hat{\varvec{x}} + i_{mn}^{y} R_{m,n}^{y}\left( \rho ,\phi \right) \hat{\varvec{y}}~, \end{aligned}$$

where $R_{m,n}$ corresponds to a given FBBF or GRBF, and $\rho$ and $\phi$ are the radial and azimuthal coordinates respectively. Those classes of basis functions are defined over the whole circular domain and have been used in^26,27^ for the analysis of the directivity of MTS antennas printed on an infinite grounded slab. One should note that both families of functions are defined in closed-form in spatial domain and admit closed-form Fourier transforms.

#### Radiated (visible) power computation

The power radiated by the MTS is derived by integrating the Poynting vector over an infinitely extended disk just above the MTS layer (x-y plane). The spectral domain electric field is denoted as $\widetilde{\mathbf{E}}(k_{x},k_{y})$, where $(k_{x},k_{y})$ are the spectral Cartesian coordinates. Using the Parseval theorem, this power can be evaluated in spectral domain as:

3 $$\begin{aligned} P_{vis}=\frac{1}{8\pi ^{2}}\int _{0}^{2\pi } \int _{0}^{k_{0}} ||\widetilde{\mathbf{E}}||^{2} ~ \frac{k_{z}}{k_{0} \eta _{0}} ~ k_{\rho } ~ dk_{\rho } ~ d\alpha , \end{aligned}$$

where $(k_{\rho },\alpha )$ are the spectral cylindrical coordinates, $k_{z}=\sqrt{k_0^2-k_{\rho }^2}$, with $k_{0}$ being the free-space wavenumber, and $\eta _{0}$ is the free-space impedance. $||\widetilde{\mathbf{E}}||$ is the norm of the spectral aperture electric field $\widetilde{\mathbf{E}}$. The latter is the sum of the electric field radiated by the current induced on the MTS and that of the feed, namely:

4 $$\begin{aligned} \widetilde{\mathbf{E}}= \underline{\underline{\widetilde{\mathbf{G}}}}^{EJ}~.~\widetilde{\mathbf{J}} + \widetilde{\mathbf{E}}_{i}~, \end{aligned}$$

where $\underline{\underline{\widetilde{\mathbf{G}}}}^{EJ}$ is the appropriate dyadic spectral Green’s function of the grounded slab and $\widetilde{\mathbf{E}}_{i}$ is the spectrum of the forced total electric field over the surface in absence of MTS. The integral in (3) can be efficiently calculated using the closed-form spectrum of the GRBFs [^26^, Expression (42)] or FBBFs [^27^, Expression (4)].

#### Surface-wave power computation

This contribution is relevant to the power of the SW that proceeds beyond the MTS. In the infinite-slab model this power is trapped in the slab, and does not contribute to radiation. However, in practical realizations it is reflected or diffracted at the slab truncation, and therefore contributes to the radiation pattern in an uncontrolled manner. For this reason, it is important to minimize this trapped SW power, during the antenna design. In the following, a closed-form expression is derived to evaluate this contribution as a function of previously computed currents. To this end, suppose that the z-component of the spectrum of the aperture electric field is a regular function that can be written as a Fourier series versus spectral angle $\alpha$:

5 $$\begin{aligned} \widetilde{E}_{z}(k_{\rho },\alpha ) = \sum _{n} \widetilde{E}_{zn}(k_{\rho })e^{-jn\alpha }. \end{aligned}$$

This form automatically comes out from (4) if GRBFs or FBBFs are used for the MTS current representation. Furthermore, the feed only contributes to the 0-indexed term, due to its axial symmetry. The spatial field is obtained by taking the inverse Fourier transform of the expression in (5). As summarized in the Appendix, for $\rho >a$, where *a* is the radius of the MTS, this can be divided into two contributions: the radiated field and the SW field. The latter is given by the contribution of the residue at the SW pole of the bare grounded slab, $\beta _0^{sw}$, and reads (see proof in the supplementary material)

6 $$\begin{aligned} {E}_{z}^{SW}=-\dfrac{j}{2}e^{-j\frac{\pi }{4}}\sqrt{\dfrac{2\beta _0^{sw}}{\pi \rho }}e^{-j\beta _0^{sw}\rho }\sum _n R_n e^{-jn\phi } , \end{aligned}$$

where we have assumed $\beta _0^{sw}\rho \gg 1$ and

7 $$\begin{aligned} R_n=\lim _{k_{\rho }\rightarrow \beta _0^{sw} } \left( k_\rho -\beta _0^{sw}\right) \widetilde{E}_{zn}=\widetilde{J}^{TM}_{n}\left( \beta _0^{sw}\right) R_{GF}+\delta _{n,0}R_{feed}~. \end{aligned}$$

In (7), $$\widetilde{J}^{TM}_{n}\left( \beta _0^{sw}\right) =\int _{0}^{2\pi }\widetilde{J}^{TM}\left( \beta _0^{sw},\alpha \right) e^{jn\alpha }d\alpha $$, $$R_{GF}$$ is the residue of the Green’s function relating the TM currents on the MTS to the vertical electric field on top of the slab, and $$ R_{feed}$$ is the residue of the field radiated by the feed on the bare slab. From (6), and taking into account the local structure of the SW electromagnetic field, the real part of the relevant Poynting vector is derived, and integrated over a cylindrical surface surrounding the radiating aperture (extending vertically from the ground plane to infinity), to obtain the following expression for the residual SW power

8 $$\begin{aligned} P_{diff}^{sw}=\dfrac{\omega \epsilon _0}{4\epsilon _r} \left[ \dfrac{\tan \left( k_{zd}h\right) }{k_{zd}}+\frac{h}{\cos ^2\left( k_{zd}h\right) }+\frac{\epsilon _r}{\alpha _{za}} \right] \sum _{n} |R_{n}|^{2}, \end{aligned}$$

where $\epsilon _{0}$ is the free-space permittivity, $\epsilon _{r}$ is the relative permittivity of the substrate, $\omega =2\pi f$ is the angular frequency, *h* is the slab thickness, $k_{zd}^2=k_0^2\epsilon _r-\left( \beta _0^{sw}\right) ^2$ and $\alpha _{za}^2=\left( \beta _0^{sw}\right) ^2-k_0^2$. Notice that the evaluation of (8) only requires the knowledge of the residues in (7), which can be calculated for any given current spectrum. The general expression can be also specialized to the particular case of a given current representation. For instance, if GRBFs are used to represent MTS currents, it results

9 $$\begin{aligned} \widetilde{J}^{TM}_{n}\left( \beta _0^{sw}\right) =\pi j^n \sum _{m=1}^{M}\left\{ \left[ j i^{x}_{m,n+1}+i^{y}_{m,n+1}\right] \Phi _{m,n+1}\left( \beta _0^{sw}\right) +\left[ i^{y}_{m,n-1}-i^{x}_{m,n-1}\right] \Phi _{m,n-1} \left( \beta _0^{sw}\right) \right\} , \end{aligned}$$

where the $\Phi$ functions are reported in [^26^, Expression (3)].

#### Power delivered by the feed

Referring to the Poynting theorem, the total power delivered by a matched feeding source in absence of losses can be computed after integrating the Poynting vector along a closed surface comprising the source. We propose to use the closed surface represented in dotted red lines in Fig. 2b. This surface includes the MTS aperture ($S_{1}$), the rim of the antenna ($S_{2}$), and the ground plane. Since the ground plane is assumed to be a perfect electric conductor, its contribution is zero. Therefore, the power delivered by the feeder can be computed as:

10 $$\begin{aligned} P_{tot}=\frac{1}{2} Re \left\{ \iint _{S_{1}} {\mathbf{E}} _{t} \times {\mathbf{H}} _{t}^{*}~.~\hat{\mathbf{n}}~dS + \iint _{S_{2}} {\mathbf{E}} \times {\mathbf{H}} ^{*}~.~\hat{\varvec{\rho }}~dS + \iiint _{V}\sigma ||{\mathbf{E}} ||^{2} dV \right\} =P_{MTS}+P_{rim}+P_{\Omega }, \end{aligned}$$

where ${\mathbf{H}}$ is the magnetic field, *t*, $*$, and *Re* stand respectively for the tangential part, the complex conjugate operator, and the real part. *V* is the volume enclosed by the surface $S_{1} \cup S_{2}$ and the ground plane. Finally, $\sigma$ is the conductivity of the dielectric substrate. The first term ($P_{MTS}$) is referred to as the MTS contribution, the second one ($P_{rim}$) as the rim contribution, and the last term ($P_{\Omega }$) is referred to as the Ohmic power losses.

#### MTS contribution

The MTS contribution is explicitly rewritten as:

11 $$\begin{aligned} P_{MTS}=\frac{1}{2} Re \left\{ \iint _{S_{1}} E_{x}~.~H_{y}^{*} - E_{y}~.~H_{x}^{*}~dS \right\} , \end{aligned}$$

where $E_{x,y}$ and $H_{x,y}$ are the Cartesian components of the electric and magnetic field respectively. The aperture tangential electric field tested with the complex conjugate of the FBBFs can be computed as:

12 $$\begin{aligned} {[}{} {\mathbf{E}} _{t}]=[{\mathbf{Z}} _{IBC}] ~ [{\mathbf{I}}], \end{aligned}$$

where $[{\mathbf{Z}} _{IBC}]$ and $[{\mathbf{I}} ]$ are respectively the impedance boundary condition (IBC) matrix and the surface current vector. The IBC matrix is calculated with the MoM procedure described in^27^. Since FBBFs are orthogonal, the bases coefficients $e_{mn}$ of $[{\mathbf{E}} _{t}]$ into FBBFs can be obtained from the tested fields $e_{mn}^{tested}$ as: $e_{mn}=e_{mn}^{tested}/K(m,n)$, where *K*(*m*, *n*) is a normalization factor [^27^, Equ. 35]. The aperture tangential magnetic field tested with FBBFs is computed as

13 $$\begin{aligned} {[}{} {\mathbf{H}}_{t}]=[{\mathbf{Z}}_{G}^{H}] ~ [{\mathbf{I}}] -[{\mathbf{V}}^{H}], \end{aligned}$$

where $[{\mathbf{Z}}_{G}^{H}]$ is the substrate matrix relating the surface current to the aperture magnetic field. $[{\mathbf{V}} ^{H}]$ is the excitation magnetic field at the MTS plane, tested with the complex conjugate of the FBBFs. $[{\mathbf{Z}}_{G}^{H}]$ and $[{\mathbf{V}}^{H}]$ are computed using a procedure similar to that used for the computation of $[{\mathbf{Z}} _{G}]$ and $[{\mathbf{V}} ]$ in^27^.

Similarly to the electric field, the magnetic field coefficients into FBBFs are obtained from the tested fields through renormalization. The aperture field is therefore written in the FBBF basis as

14 $$\begin{aligned}&{\mathbf{E}} _{t}(\rho ,\phi )= \sum _{n=-N}^{N} \sum _{m=1}^{M} e_{mn}^{x} R_{m,n}^{x}(\rho ,\phi ) \hat{\varvec{x}} + e_{mn}^{y} R_{m,n}^{y}(\rho ,\phi ) \hat{\varvec{y}} \end{aligned}$$

15 $$\begin{aligned}&{\mathbf{H}} _{t}(\rho ,\phi )= \sum _{n=-N}^{N} \sum _{m=1}^{M} h_{mn}^{x} R_{m,n}^{x}(\rho ,\phi ) \hat{\varvec{x}} + h_{mn}^{y} R_{m,n}^{y}(\rho ,\phi ) \hat{\varvec{y}}. \end{aligned}$$

Inserting expressions (14) and (15) into (11), and using the orthogonality relation of the FBBFs (see [27, Appendix A]), the MTS contribution to the delivered power is given by

16 $$\begin{aligned} P_{MTS}=\frac{1}{2}\sum _{n=-N}^{N} \sum _{m=1}^{M} K(m,n) Re\left\{ e_{mn}^{x} h_{mn}^{y*} - e_{mn}^{y} h_{mn}^{x*}\right\} . \end{aligned}$$

This means that the calculation of the MTS contribution to the power delivered by the feed can be carried out extremely fast (in practice in less than 1 min) once the currents on the MTS are known in term of FBBFs (e.g. using the MoM).

#### Rim contribution

The rim contribution in (10) can be developed as

17 $$\begin{aligned} P_{rim}=\frac{a}{2} Re \left\{ \int _{z=-h}^{0} \int _{\phi =0}^{2\pi } E_{\phi }(\rho =a,\phi ,z) ~ H_{z}^{*}(\rho =a,\phi ,z) -E_{z}(\rho =a,\phi ,z) ~ H_{\phi }^{*}(\rho =a,\phi ,z) ~d\phi ~dz \right\} . \end{aligned}$$

Since the mode is quasi-TM over a ground plane, it is reasonable to neglect the first term. In the following, we will hence detail the calculation of the second term only; bearing in mind that the first term can be computed similarly.

18 $$\begin{aligned} {\begin{matrix} P_{rim} \approx -\frac{a}{2} Re \left\{ \int _{z=-h}^{0} \int _{\phi =0}^{2\pi } E_{z}(\rho =a) ~ H_{\phi }^{*}(\rho =a) ~d\phi ~dz\right\} , \end{matrix}} \end{aligned}$$

where the arguments $\phi$ and *z* have been omitted. Expression (18) can be rewritten as

19 $$\begin{aligned} P_{rim}\approx \frac{a}{2} Re \left\{ \iint E_{z}(\rho =a) ~ H_{x}^{*}(\rho =a) \sin \phi ~d\phi ~dz\right\} - \frac{a}{2} Re \left\{ \iint E_{z}(\rho =a) ~ H_{y}^{*}(\rho =a) \cos \phi ~d\phi ~dz\right\} . \end{aligned}$$

Now, the $E_{z}$ field is split into three contributions $E_{zx}$, $E_{zy}$, and $E_{zz}$, corresponding respectively to the *x*-directed surface current, *y*-directed surface current and the *z*-directed excitation current. In the same way, we define $H_{xx}$, $H_{xy}$, $H_{xz}$, $H_{yx}$, $H_{yy}$, and $H_{yz}$. The spatial field evaluation at the antenna radius, is carried out by computing with a complex-contour deformation, the relevant Sommerfeld integrals with the appropriate Green’s functions. Those integrands converge relatively fast, given the narrow bandwidth of the FBBF spectrum. However, when evaluating the feeding contribution, at (or close to) the feeding layer, the integrand does not converge well. In this case, one should extract an asymptotic term corresponding to the homogenous medium Green’s function while adding this term explicitly in spatial domain. This procedure is explained in the supplementary material.

To efficiently compute azimuthally the rim contributions, the key issue consists of expressing the azimuthal variation of the fields in (19) through Fourier harmonics. Then, using Parseval’s identity, one can compute the power in (19) as a single summation over the harmonics. Integration along *z* is carried out numerically, with a few sampling points (about 4). Details regarding the fields expansion procedure into Fourier Harmonics can be found in the supplementary material as well as in Ref.^31^, Section 7.3.2.

#### Ohmic power losses

This section is devoted to the Ohmic power losses evaluation in the MTS substrate. It is assumed that the losses in the metallization are negligible compared to the substrate loss, which is a good assumption at frequencies lower than 30 GHz^29^. Considering a substrate with electric conductivity $\sigma$, the Ohmic power loss is given by the third term in (10), where the integration is carried out over the volume just beneath the MTS of radius *a*, over a height corresponding to the substrate thickness *h*. In order to efficiently compute the electric field in the substrate, we have defined a substrate interaction matrix $[{\mathbf{Z}} _{G}^{S}(z)]$ between the surface current ${\mathbf{J}}$ and the scattered electric field in a given planar layer in the substrate (fixed height *z*) of radius *a*. The interaction matrix $[{\mathbf{Z}}_{G}^{S}(z)]$, as well as the excitation fields vector $[{\mathbf{V}} ^{E}(z)]$, can be computed in terms of FBBFs using a procedure similar to what has been described in^27^. Therefore, the total electric field at a given height *z* in the substrate, tested with the complex conjugate of the FBBFs $[{\mathbf{E}} (z)]$ can be calculated as

20 $$\begin{aligned} {[}{} {\mathbf{E}} (z)]=[{\mathbf{Z}}_{G}^{S}(z)] ~ [{\mathbf{I}} ] -[{\mathbf{V}} ^{E}(z)]~. \end{aligned}$$

Based on (20), the coefficients $e_{mn}$ of the total electric field in FBBFs are derived from the tested fields through renormalization. After expanding each component of the electric field into FBBFs and using the orthogonality relation of the FBBFs, the Ohmic power loss in (10) can be rewritten as:

21 $$\begin{aligned} P_{\Omega }= \frac{\sigma }{2} \sum _{m,n}K(m,n) \int _{z=-h}^{0} ( e_{mn}^{x*}e_{mn}^{x} + e_{mn}^{y*}e_{mn}^{y} + e_{mn}^{z*}e_{mn}^{z} ) ~ dz~, \end{aligned}$$

which means that the Ohmic power losses can be computed extremely fast assuming that the fields expansion coefficients are known. The computation time for the fields coefficients is dominated by the computation time of the underlying substrate matrix $[{\mathbf{Z}} _{G}^{S}(z)]$, which requires in practice a few seconds (per sampled layer in the substrate) on a traditional laptop computer.

### Efficiencies under consideration

From the previous sections, the total power delivered by the feed can be calculated in two different ways i.e

22 $$\begin{aligned} P_{tot}= P_{rim} + P_{MTS} +P_{\Omega }=P_{vis}+P_{diff}^{sw}+P_{\Omega }~. \end{aligned}$$

The first method is based on FBBFs and the second one uses GRBFs. It is worth mentioning that although the second method is implemented with GRBFs, this approach can also be used with any kind of basis functions. Now, we define the following efficiencies.

#### Conversion efficiency

The conversion efficiency $\epsilon _{conv}$ is defined as the ratio between the radiated power $P_{vis}$ and the total power delivered by the feed

23 $$\begin{aligned} \epsilon _{conv}= \frac{P_{vis}}{P_{tot}}. \end{aligned}$$

Note that the radiated power is computed taking also into account the space waves directly radiated by the feed. Therefore, the conversion efficiency is only affected by the losses and the reflection/diffraction effects at the substrate rim. In contrast to what is defined in^29^, the conversion efficiency is defined on the basis of the total radiated power, i.e. the feed efficiency is not separately treated here.

#### Tapering efficiency

The tapering (aperture) efficiency $\epsilon _{tap}$ is the ratio between the antenna directivity and the maximum directivity that can be achieved considering the antenna area. The maximum directivity is obtained with a uniform illumination of the aperture. For a broadside beam, the tapering efficiency is

24 $$\begin{aligned} \epsilon _{tap}= \frac{\lambda ^2 D_{MAX}}{4\pi S_{1}}, \end{aligned}$$

where $S_{1}=\pi a^{2}$ is the physical antenna area, $\lambda$ is the free-space wavelength, and $D_{MAX}$ is the maximum antenna directivity. Note that the directivity is computed assuming an infinitely extended substrate. Hence, contrary to the conversion efficiency, diffraction effects originating from the truncation of the substrate are not taken into account in the tapering efficiency.

#### Loss factor

The loss factor $L_{loss}$ quantifies the impact of the losses on the conversion efficiency. It is defined as:

25 $$\begin{aligned} L_{loss}= \frac{P_{\Omega }}{P_{tot}}. \end{aligned}$$

A small loss factor means that the conversion efficiency is mainly limited by the diffraction effects at the substrate rim.

#### Diffraction factor

The diffraction factor $L_{diff}$ evaluates the impact of the substrate truncation effects on the conversion efficiency. More precisely, it quantifies the power lost in SW’s propagating beyond the MTS in the assumption of an infinite grounded dielectric substrate.

26 $$\begin{aligned} L_{diff}= \frac{P_{diff}^{sw}}{P_{tot}}=\frac{P_{rim} + P_{MTS} - P_{vis}}{P_{tot}}. \end{aligned}$$

A small diffraction factor means that the loss factor plays a major role in the value of conversion efficiency.

#### Compound efficiency

There is no universal figure of merit to define the efficiency of an antenna, since the compromise between the previous metrics will depend on the application of interest. In this paper, we define a “compound efficiency” as the product of the conversion efficiency with the tapering efficiency:

27 $$\begin{aligned} \epsilon =\epsilon _{tap}~\epsilon _{conv}. \end{aligned}$$

This choice is roughly justified by the fact that a zero (or very small) value of the tapering/conversion efficiency automatically leads to a zero (or very small) compound efficiency.

### Numerical and experimental validations

This section provides numerical and experimental validations of the efficiency computation methods presented in the previous sections.

#### Isotropic MTS

First, we consider a broadside beam MTS antenna operating at 17 GHz, modeled as an impenetrable scalar impedance given by^26^

28 $$\begin{aligned} \text {Z}^\text {+}(\rho ,\phi ) = j X_0 \left[ 1 + M \sin (2 \pi \rho /p - \phi ) \right] , \end{aligned}$$

where the average reactance is $X_{0}=0.71~\eta _{0}$, with $\eta _{0}$ being the free-space impedance. The period *p* is defined as $p=\lambda /\sqrt{1+(X_{0}/\eta _{0})^{2}}$, with $\lambda$ being the free-space wavelength. *M* is the modulation index (depth). The MTS radius is fixed to $a=5.65\lambda$ and the sheet impedance lays on the substrate ROGERS 4350B of relative permittivity $\epsilon _{r}=3.66$, and thickness $h=1.524$ mm. The antenna is fed at its center with a vertical elementary dipole placed in the middle of the substrate ($\rho =0$, $z=-h/2$).

**Figure 3:**
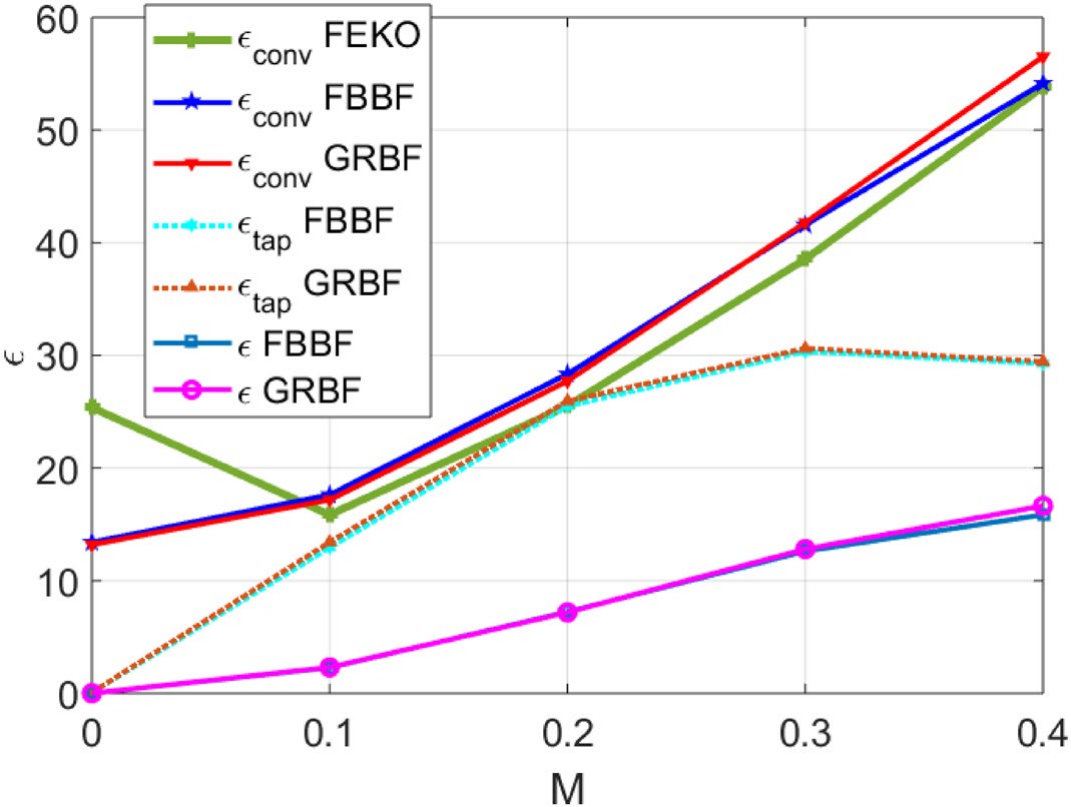
Efficiency (in $\%$) of an isotropic MTS antenna radiating a broadside pencil beam.

Figure 3 illustrates the conversion and tapering efficiencies versus modulation index *M*, computed with the FBBF and the GRBF based methods. The conversion efficiency obtained with the commercial software FEKO^32^ is reported in the same figure. An excellent agreement is observed between the three approaches, despite being based on completely different basis functions (FEKO uses Rao-Wilton-Glisson (RWG)^33^ basis functions) and efficiency computation methods. At $M=0$, there is a mismatch with FEKO probably due to a different feed modeling. One should note that the FBBF and the GRBF based methods require respectively 2.5 min and 1 min of total computation time on a conventional laptop computer, while FEKO needs 2.4 h. As expected, for relatively low modulation depths, the conversion efficiency is small. However, the conversion efficiency is not zero at $M=0$ due to the space waves directly radiated by the feed. Moreover, the tapering efficiency is also relatively small for small *M* because the tapering efficiency takes into account the space waves from the feeder. Indeed, for small modulation depths, the radiation contribution is dominated by those space waves, which are not present at broadside because the feeder is a TM vertical dipole. Increasing the modulation depth, the conversion efficiency improves, but the tapering efficiency reaches a maximum at approximately $M=0.3$. For higher values of *M*, the tapering efficiency starts to decrease because the uniformity of the tapering illumination rapidly degrades.

#### Anisotropic MTS with uniform modulation depth

**Figure 4:**
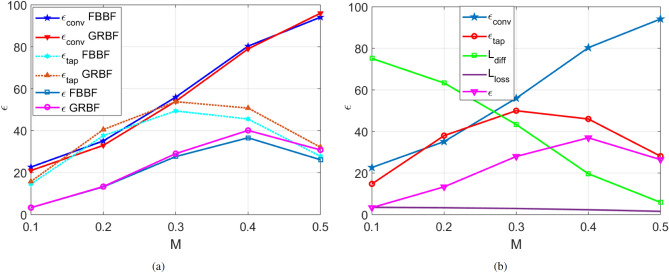
Efficiency (in $\%$) of an anisotropic MTS antenna radiating a broadside pencil beam. (**a**) In absence of Ohmic losses. (**b**) With Ohmic losses in the substrate and using the FBBF based method.

A broadside beam anisotropic MTS antenna implementing a sheet impedance modulation is now considered.

29 $$\begin{aligned} \begin{aligned}{}&\text {Z}^{\rho \rho }(\rho ,\phi ) = j X_0 \left[ 1 + M(\rho ) \cos (\beta _{sw} \rho - \phi ) \right] \\&\text {Z}^{\rho \phi }(\rho ,\phi ) = j X_0 M(\rho ) \sin (\beta _{sw} \rho - \phi )\\&\text {Z}^{\phi \phi }(\rho ,\phi ) = j X_0 \left[ 1 - M(\rho ) \cos (\beta _{sw} \rho - \phi ) \right] , \end{aligned} \end{aligned}$$

where $X_0=-377 ~ \Omega$, and $\beta _{sw}$ is the SW wavenumber supported by a uniform sheet with reactance $X_0$ laying on a grounded substrate of relative permittivity $\epsilon _{r}=3$ and thickness $h=0.762$ mm. The antenna operates at 29.75 GHz with a radius equal to 13.6 cm. Figure 4a shows the efficiencies obtained with the two methods. A very good agreement between the FBBF and the GRBF based methods is observed. The small difference in the aperture efficiency could be traced back to differences in surface current modeling close to the feed region. GRBFs exclude in the current the portion of the MTS occupied by the feed, while FBBFs consider the current distribution on the whole circular domain. A detailed discussion regarding this aspect can be found in^27^. Anyway, a trend similar to that of the isotropic MTS is observed. However, the tapering efficiency is better in comparison with the isotropic MTS case owing to a better polarization purity^11^. The overall (compound) efficiency is maximum approximately at $M=0.4$ and is approximately equal to $40\%$. That means, a good compromise should be found between the tapering efficiency and the conversion efficiency. Indeed, a relatively low modulation depth will lead to strong reflection/diffraction effects at the substrate rim which are not predicted by the MoM simulation tools (based on infinite substrate assumption). This diffraction will increase the measured sidelobe level. On the contrary, a high modulation depth will considerably degrade the tapering efficiency of the antenna. Therefore, the methods developed in this paper are useful for the antenna designer to rapidly and accurately optimize the efficiency of the antenna without the need of huge computational resources. Those tools may also be used with MTS antennas designed with the traditional holographic technique^7^; in that case, it provides a rapid way to rigorously check the power balance obtained with holographic theory.

Now, losses are introduced in the substrate through a dissipation factor $\tan \delta =0.001$. The trend of the conversion efficiency, tapering efficiency, diffraction factor and losses factor is reported in Fig. 4b. One can conclude that the losses are very small (about $(2-3)\%$ of the power delivered by the feeder) and the impact of the losses on the compound efficiency is negligible. This is one of the main advantages of this class of antennas.

#### Anisotropic MTS with non uniform modulation depth

**Figure 5:**
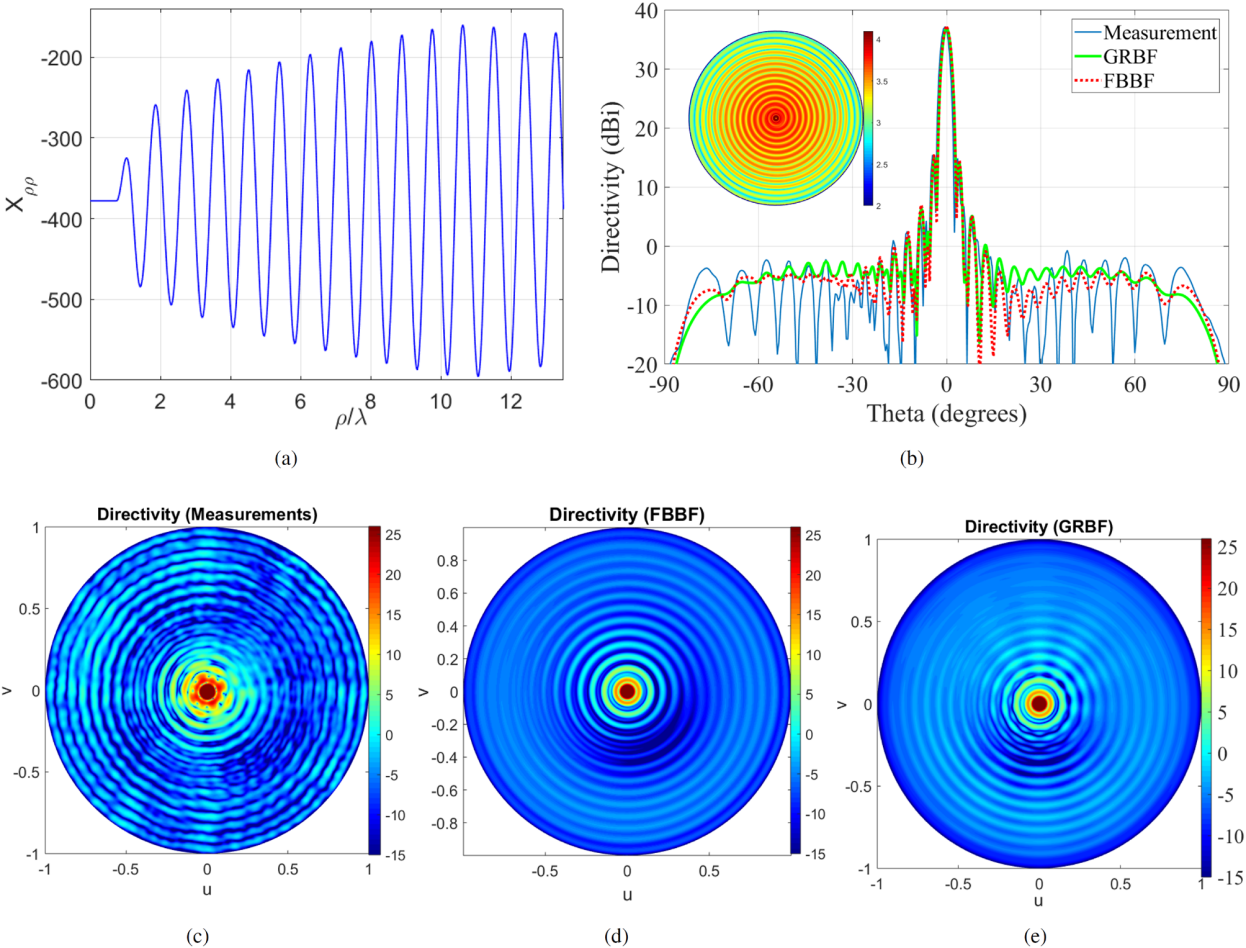
Anisotropic MTS antenna with non-uniform modulation index. (**a**) $X_{\rho \rho }$ reactance at $\phi =0$. (**b**) Directivity at $\phi =0$. The inset disk represents the absolute value of the current distribution on the MTS in log scale. (**c**) Measured directivity (dBi) in the uv plane. (**d**) Simulated directivity (dBi) in the uv plane with FBBF. (**e**) Simulated directivity (dBi) in the uv plane with GRBF. The conversion efficiency of the antenna is about $95\%$ with a $70\%$ compound efficiency.

The previous example shows that one can reach $40\%$ compound efficiency by optimizing the value of the uniform modulation depth. However, better performance can be achieved by impressing a non-uniform modulation depth^18^. This means that the modulation index increases with respect to the radial coordinate so as to maintain a quasi-uniform aperture field illumination (see Fig. 5a). Such antenna has been designed in^18^ using the FO formalism^30^. Details regarding the design and measurements can be found in^18^. The analysis of this MTS provides (with the two methods proposed in this paper) a conversion efficiency of about $95\%$, which means that almost all the power delivered by the feed is radiated by the antenna. Diffraction at the antenna rim can therefore be neglected. As a result, the directivity analysis with the infinite substrate assumption should provide a good estimate of the measured directivity. Figure 5b compares a cut of the copolar directivity computed using the MoM codes based on FBBF and GRBF with the measurements. The 3D copolar radiation pattern is represented in Fig. 5c–e. One can observe a very good agreement between measurements and simulations. The slight difference in the far sidelobes can be explained by the residual SW power diffracted at the antenna rim, the IBC implementation process, and the modeling of the feeder. The latter is modeled as a simple infinitesimal dipole. Nevertheless, the maximum directivity (about 37.11 dBi) is well predicted by the MoM tools based on GRBF and FBBF, due to the very high conversion efficiency. Lower conversion efficiencies will lead to a higher (in comparison with measurements) predicted maximum directivity. The measured and simulated directivity of the antenna corresponds to about $70\%$ compound efficiency. This efficiency can exceed $80\%$ by minimizing the space waves power directly radiated by the feed. This calls for an efficient SW launcher.

## Discussion

Two algorithms have been proposed for the fast evaluation of the efficiency of MTS antennas. Those tools may be used with antenna designs that have been obtained using any type of MTS synthesis, including the traditional holographic method^7^, or the more accurate Flat optics^19^, or the direct inversion method^21^. Whenever the antenna is synthesized, the two formulations presented here provide a rapid algorithm to rigorously check the power balance provided by any of the above mentioned methods. The first formulation is based on the integration of the Poynting vector along a surface enclosing the feed while the second approach relies on the residue evaluation of the electric field at the substrate SW pole. Those algorithms can cope with arbitrary anisotropic surface impedance modulation and provide accurate results in a few minutes. Although based on completely different formalisms and basis functions, those methods provide approximately the same efficiency results and those results have been confirmed with commercial software and experimental data. The method based on the residue theorem is more analytical and, hence, faster than the Poynting vector based method. Conversely, the Poynting vector based method may be applied to a more general class of problems. Progressive improvement of the antenna compound efficiency is illustrated from isotropic MTS with uniform modulation index ($20\%$ efficiency) to anisotropic MTS with optimized (non-uniform) modulation index profile ($70\%$ efficiency). To reach and exceed $80\%$ efficiency, one needs to properly design the feeder so as to minimize the space waves directly radiated by the feeder. The study also demonstrated that the Ohmic power losses in modulated MTS antennas are very small ($< 10\%$ of the total power) at frequencies lower than 30 GHz. At higher frequencies, one should consider the losses in the metallization. Finally, as in the present paper, the feeder has been modeled as an infinitesimal monopole, the reflection coefficient of the antenna cannot be computed. An accurate estimate of the reflection coefficient should require a full-wave analysis of the MTS including details regarding the feeder.

## Methods

### FBBF and GRBF analysis

Each FBBF and GRBF is defined according to two parameters *m* and *n* with $m=1~\ldots ~M$ and $n=-N~\ldots ~N$ (see equation (2)). The total (x-directed and y-directed) number of basis functions is therefore $2M(2N+1)$. For the first example, isotropic MTS, we used for FBBF and GRBF $N=8$ and $M=46$, which corresponds to 1564 basis functions. In the anisotropic MTS examples, the MTS has been analyzed with FBBF using $N=10$ and $M=80$ i.e. 3360 FBBFs, and with GRBF using $N=10$ and $M=90$ i.e. 3780 GRBFs.

### FEKO analysis

**Figure 6:**
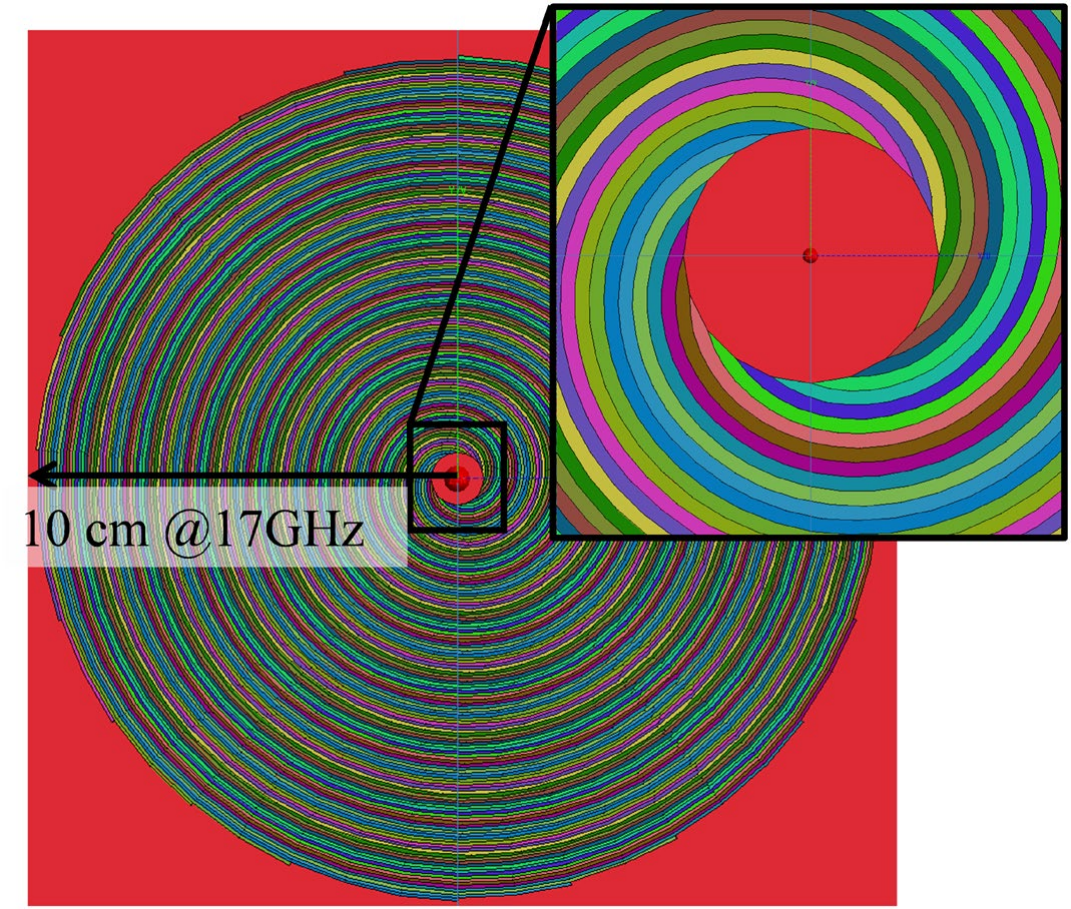
A FEKO simulation setup, each spiral strip of different color stands for a different value of constant surface reactance.

FEKO implements the EFIE and allows one to use layered medium Green’s functions (FEKO’s GF card) as with FBBF and GRBFs. The FEKO simulations have been performed using the impedance boundary condition (IBC) available in this software (SK card and user defined surface impedance). Hence, all the results have been obtained using the same IBC and integral equation, for a fair comparison. Figure 6 shows a top view of the simulated structure. Each color strip with spiral shape has a width equal to one tenth of the free space wavelength, and it corresponds to a region with a constant isotropic reactance. Thus, the spiral strips (see Fig. 6) are not used as discrete elements, but to separate the antenna aperture in regions with a constant IBC, i.e., each spiral strip corresponds to a value of surface reactance. Moreover, the structure has been excited using an electric Hertzian dipole (A5 card in FEKO) placed at the origin, in the middle of the dielectric slab. The EFIE currents and radiated fields have been obtained using the standard FEKO solver with 54907 RWG basis functions.

## Supplementary information

Supplementary material 1
