# Supplementary material for: Power balance and efficiency of metasurface antennas
Source: Sci Rep. 2020 Oct 15;10:17508. doi: 10.1038/s41598-020-74674-w (PMC7567091; doi:10.1038/s41598-020-74674-w)
Supplement: Supplementary file 1 — Supplementary material 1 [file 41598_2020_74674_MOESM1_ESM.pdf]

Supplementary information to the manuscript entitled: Power Balance and Efficiency of Metasurface Antennas

Modeste Bodehou[1,*], David González-Ovejero[2], Christophe Craeye[1], Stefano Maci[3], Isabelle Huynen[1], and Enrica Martini[3]

[1]ICTEAM institute, Université catholique de Louvain, Place du Levant 3, 1348 Louvain-la-Neuve, Belgium.
[2]Univ Rennes, CNRS, Institut d'Electronique et de Télécommunications de Rennes (IETR), UMR 6164, 35000 Rennes, France.
[3]Department of Information Engineering and Mathematics, University of Siena, 53100 Siena, Italy.
[*]modeste.bodehou@uclouvain.be

ABSTRACT

This supplementary information provides the derivation of the SW field contribution from the vertical electric field spectrum $\widetilde{E}_z(k_\rho,\alpha)$ and details regarding the rim power contribution.

Surface-wave field evaluation

The total spatial field is first expressed as the inverse Fourier transform of the spectrum

$$E_z(\rho,\phi) = \frac{1}{4\pi^2}\int_0^\infty \int_0^{2\pi} \widetilde{E}_z(k_\rho,\alpha) e^{-jk_\rho(cos\alpha\cos\phi+\sin\alpha\sin\phi)} d\alpha k_\rho dk_\rho = \frac{1}{2\pi}\sum_n (-j)^n e^{-jn\phi}\int_0^\infty \widetilde{E}_{zn}(k_\rho) J_n(k_\rho\rho) k_\rho dk_\rho, \quad (1)$$

where J_n is the first kind Bessel function of order n. The integral in k_ρ is then cast in the following alternative form

$$\int_0^\infty \widetilde{E}_{zn}(k_\rho) J_n(k_\rho\rho) k_\rho dk_\rho = \frac{1}{2}\int_{-\infty}^{+\infty} \widetilde{E}_{zn}(k_\rho) H_n^{(2)}(k_\rho\rho) k_\rho dk_\rho, \quad (2)$$

where the contour of integration C_1 is defined so as to avoid the branch cut of the Hankel function along the negative imaginary axis, as shown in Fig. 1 and the result has been obtained by exploiting the following properties of the functions $\widetilde{E}_{zn}$, which are a consequence of the supposed regularity of $\widetilde{E}_z$:

- $\widetilde{E}_{zn}$ has a zero at least of order n at $k_\rho = 0$
- $\widetilde{E}_{zn}$ is an even (odd) function of k_ρ for n even (odd).

In order to extract the SW field from the integral in (2), we deform the integration path to the new contour C_2, shown in blue in Fig. 1. Note that the integrand function has two branch points at $k_\rho = \pm k_0$ and a pole at $k_\rho = \beta_0^{sw}$, considered to be complex numbers in order to make them lie off the real k_ρ axis. The integrand is analytic along $C_1 + C_2$, but the pole β_0^{sw} is captured during the deformation from C_1 to C_2. From the Residue theorem, the integral in (2) can therefore be written as the summation of the integral along C_2, which provides the space-wave, and the residue contribution, corresponding to the SW field. This latter is therefore

$$E_z^{sw}(\rho,\phi) = \frac{\beta_0^{sw}}{2}\sum_n (-j)^{n+1} e^{-jn\phi} R_n H_n^{(2)}(\beta_0^{sw}), \quad (3)$$

After substituting the asymptotic expression of the Hankel function for large argument, one obtains

$$E_z^{SW} = -\frac{j}{2} e^{-j\frac{\pi}{4}} \sqrt{\frac{2\beta_0^{sw}}{\pi\rho}} e^{-j\beta_0^{sw}\rho} \sum_n R_n e^{-jn\phi}. \quad (4)$$

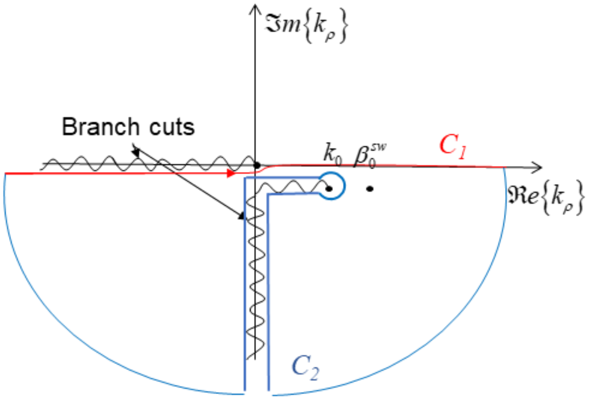

Figure 1. Integration path in the complex k_ρ plane.

Rim power contribution

Evaluation of E_{zx}

The electric field E_{zx} can be computed in spectral domain based on the x-directed current distribution (assumed to be known in Fourier-Bessel basis) and the pertaining spectral Green's function $\widetilde{G}_{zx}^{EJ}$. One then needs to calculate the inverse Fourier transform of $\widetilde{E}_{zx}$, which can be written as:

$$E_{zx} = \frac{1}{4\pi^2} \sum_{m,n} i_{mn}^{x} \iint \widetilde{G}_{zx}^{EJ} \widetilde{R}_{mn} e^{-j(k_x x + k_y y)} dk_x dk_y \tag{5}$$

The spectral Green's function $\widetilde{G}_{zx}^{EJ}$ and the FBBF spectrum $\widetilde{R}_{mn}$ can be respectively written in the form $\widetilde{g}_{zx}^{E}(k_\rho)\cos\alpha$, and $\widetilde{F}_{mn}(k_\rho)e^{-jn\alpha}$. After inserting the latter expressions in (5), and going through cylindrical coordinates ($k_x = k_\rho \cos\alpha,\ k_y = k_\rho \sin\alpha$), expression (5) can be rewritten as

$$E_{zx} = \frac{1}{4\pi^2} \sum_{m,n} i_{mn}^{x} \int_0^\infty \widetilde{g}_{zx}^{E}\, \widetilde{F}_{mn} \int_0^{2\pi} \cos\alpha\; e^{-jn\alpha} e^{-jk_\rho\rho\cos(\phi-\alpha)}\, d\alpha\; k_\rho\; dk_\rho \tag{6}$$

Integration along α can be carried out in closed form, leading to:

$$E_{zx} = \frac{1}{4\pi^2} \sum_{m,n} i_{mn}^{x} \int_0^\infty \widetilde{g}_{zx}^{E}\, \widetilde{F}_{mn}\; j \left[f_n^0(k_\rho\rho,\phi,1) - f_n^0(k_\rho\rho,\phi,-1) \right] k_\rho\; dk_\rho \tag{7}$$

where the function f_n^0 is defined as

$$f_n^0(k_\rho\rho,\phi,i) = \pi j^n e^{-j(n+i)\phi} J_{n+i}(-k_\rho\rho) \tag{8}$$

and $J_n(x)$ denotes the first kind Bessel function of order n. To simplify the notation, we define the functions:

$$f_{mn}^{a}(k_\rho\rho,\phi,-1) = -j\; f_n^0(k_\rho\rho,\phi,-1)\; \widetilde{g}_{zx}^{E}\; \widetilde{F}_{mn} \tag{9}$$

and

$$f_{mn}^{b}(k_\rho\rho,\phi,1) = j\; f_n^0(k_\rho\rho,\phi,1)\; \widetilde{g}_{zx}^{E}\; \widetilde{F}_{mn}. \tag{10}$$

Then, according to (9), and (10), E_{zx} is rewritten as

$$E_{zx} = \frac{1}{4\pi^2} \sum_{m,n} i_{mn}^{x} \int_0^\infty \left[f_{mn}^{a}(k_\rho\rho,\phi,-1) + f_{mn}^{b}(k_\rho\rho,\phi,1) \right] k_\rho\; dk_\rho \tag{11}$$

Evaluation of E_{zy}

Following a similar reasoning, E_{zy} is given by:

$$E_{zy} = \frac{1}{4\pi^2}\sum_{m,n} i^y_{mn}\int_0^\infty \widetilde{g}^E_{zy}\,\widetilde{F}_{mn}\left[-f^0_n(k_\rho\rho,\phi,-1)\ -f^0_n(k_\rho\rho,\phi,1)\right]k_\rho\,dk_\rho \tag{12}$$

Note that $\widetilde{g}^E_{zy} = \widetilde{g}^E_{zx}$. After defining the following functions:

$$f^c_{mn}(k_\rho\rho,\phi,-1) = -f^0_n(k_\rho\rho,\phi,-1)\,\widetilde{g}^E_{zy}\,\widetilde{F}_{mn} \tag{13}$$

$$f^d_{mn}(k_\rho\rho,\phi,1) = -f^0_n(k_\rho\rho,\phi,1)\,\widetilde{g}^E_{zy}\,\widetilde{F}_{mn} \tag{14}$$

E_{zy}, is written in compact form as:

$$E_{zy} = \frac{1}{4\pi^2}\sum_{m,n} i^y_{mn}\int_0^\infty \left[f^c_{mn}(k_\rho\rho,\phi,-1)+\ f^d_{mn}(k_\rho\rho,\phi,1)\right]k_\rho\,dk_\rho \tag{15}$$

Evaluation of E_{zz}

Since the MTS is assumed to be fed with a vertical elementary dipole placed at the center ($\rho = 0$), the z-component of the excitation electric field does not depend on the azimuthal direction. Therefore, $\widetilde{G}^{EJ}_{zz}$ can be written as $\widetilde{g}^E_{zz}(k_\rho)$. Then, E_{zz} is computed as:

$$E_{zz} = \frac{i^z_{00}}{4\pi^2}\iint \widetilde{g}^E_{zz}\,e^{-jk_\rho\rho\cos(\phi-\alpha)}\,d\alpha\,k_\rho\,dk_\rho \tag{16}$$

where i^z_{00} is the complex excitation amplitude. After integrating along α, we get

$$E_{zz} = \frac{i^z_{00}}{4\pi^2}\int_0^\infty f^z_{00}(k_\rho\rho,\phi,0)\,k_\rho\,dk_\rho \tag{17}$$

with

$$f^z_{00}(k_\rho\rho,\phi,0) = 2f^0_0(k_\rho\rho,\phi,0)\,\widetilde{g}^E_{zz} \tag{18}$$

Evaluation of H_{xx}

The x-directed magnetic field Green's function associated to a x-directed current distribution can be written spectrally as $\widetilde{G}^{HJ}_{xx} = \cos\alpha\ \sin\alpha\,\widetilde{g}^H_{xx}(k_\rho)$. The magnetic field in spatial domain is then derived as

$$H_{xx} = \frac{1}{4\pi^2}\sum_{m,n} i^x_{mn}\int_0^\infty \widetilde{g}^H_{xx}\,\widetilde{F}_{mn}\,\frac{j}{2}\left[f^0_n(k_\rho\rho,\phi,-2)\ -f^0_n(k_\rho\rho,\phi,2)\right]k_\rho\,dk_\rho \tag{19}$$

Similarly to the previous cases, we defined:

$$f^e_{mn}(k_\rho\rho,\phi,-2) = \frac{j}{2}f^0_n(k_\rho\rho,\phi,-2)\,\widetilde{g}^H_{xx}\,\widetilde{F}_{mn} \tag{20}$$

and

$$f^f_{mn}(k_\rho\rho,\phi,2) = -\frac{j}{2}f^0_n(k_\rho\rho,\phi,2)\,\widetilde{g}^H_{xx}\,\widetilde{F}_{mn} \tag{21}$$

Expression (19) is then written compactly as follows

$$H_{xx} = \frac{1}{4\pi^2}\sum_{m,n} i^x_{mn}\int_0^\infty \left[f^e_{mn}(k_\rho\rho,\phi,-2)+\ f^f_{mn}(k_\rho\rho,\phi,2)\right]k_\rho\,dk_\rho \tag{22}$$

Evaluation of H_{xy}, H_{yx}, H_{yy}, H_{xz}, and H_{yz}

The x-directed magnetic field spectral Green's function corresponding to a y-directed current takes the form $\widetilde{G}^{HJ}_{xy} = \widetilde{g}^H_{xy1}(k_\rho)\cos^2\alpha + \widetilde{g}^H_{xy2}(k_\rho)\sin^2\alpha$. Therefore, the magnetic field can be written as the sum of two contributions, namely H_{xy1} and H_{xy2} corresponding to each term of the Green's function. Those contributions as well as the other magnetic fields components are computed in a manner similar to that of the previous fields.

Computation of the rim contribution

Let us consider the first term in the rim power contribution, namely P_{rim1}, given by

$$P_{rim} = \frac{1}{2} Re \left\{ \iint E_z(\rho = a)\, H_x^*(\rho = a) \sin\phi \; d\phi \; dz \right\} \tag{23}$$

The method is explained for the first term in the rim contribution. The second term is computed similarly.

The computation of E_z and H_x has been previously explained and written compactly as:

$$E_z = \frac{1}{4\pi^2} \int_0^\infty \left\{ \sum_{m,n} \left[i_{mn}^x \left(f_{mn}^a(k_\rho \rho, -1) + f_{mn}^b(k_\rho \rho, 1) \right) \; + i_{mn}^y \left(f_{mn}^c(k_\rho \rho, -1) + f_{mn}^d(k_\rho \rho, 1) \right) \right] \; + i_{00}^z f_{00}^z(k_\rho \rho, 0) \right\} k_\rho \; dk_\rho \tag{24}$$

$$\begin{aligned} H_x = \frac{1}{4\pi^2} \int_0^\infty \Bigg\{ \sum_{m,n} \Big[& i_{mn}^x \left(f_{mn}^e(k_\rho \rho, -2) + f_{mn}^f(k_\rho \rho, 2) \right) \; + i_{mn}^y \Big(f_{mn}^g(k_\rho \rho, 0) + f_{mn}^h(k_\rho \rho, -2) + f_{mn}^i(k_\rho \rho, 2) \\ & + f_{mn}^j(k_\rho \rho, 0) + f_{mn}^k(k_\rho \rho, -2) + f_{mn}^l(k_\rho \rho, 2) \Big) \Big] + i_{00}^z f_{00}^{x1}(k_\rho \rho, -1) + i_{00}^z f_{00}^{x2}(k_\rho \rho, 1) \Bigg\} k_\rho \; dk_\rho \end{aligned} \tag{25}$$

where the argument ϕ in the functions $f_{mn}^{()}$ has been omitted. The summation along the variable m in (24) and (25) can be precomputed. This allows writing the fields as a sum on n only, which corresponds to an harmonic representation of the fields along the azimuth ϕ. Integration along ϕ in (23) can therefore be carried out analytically after pre-computing integrals along k_ρ in (24) and (25).

Asymptotic component extraction for the excitation fields evaluation

When evaluating integrals along k_ρ in (17) at (or very close to) the excitation dipole layer ($z = -h/2$, with h being the substrate thickness), the integrand does not converge rapidly. In this case, accurate results can be obtained by subtracting from the integrand a spectral asymptotic term corresponding to the homogeneous medium Green's function and adding the same contribution in closed space-domain form. As an example, one needs to calculate:

$$E_{zz} = \frac{i_{00}^z}{2\pi} \int_0^\infty J_0(k_\rho a)\; \tilde{g}_{zz}^E \; k_\rho \; dk_\rho \tag{26}$$

Integral (26) is reformulated as

$$E_{zz} = \frac{i_{00}^z}{2\pi} \int_0^\infty J_0(k_\rho a)\; . \; \left[\tilde{g}_{zz}^E - \tilde{g}_{zz}^{Ehom} \right] k_\rho \; dk_\rho + i_{00}^z E_{zz}^{hom} \tag{27}$$

where $\tilde{g}_{zz}^{Ehom}$ is zz the spectral Green's function in the homogeneous medium of relative permittivity corresponding to that of the MTS substrate, and E_{zz}^{hom} is the spatial Green's function of the homogeneous medium given by:

$$E_{zz}^{hom} = i_{00}^z \frac{e^{-jkr}}{4\pi r} \left[-j\; \eta\; k\; \sin^2\theta \; \left(1 - \frac{j}{kr} - \frac{1}{(kr)^2} \right) + \frac{2\eta}{r} \cos^2\theta \left(1 - \frac{j}{kr} \right) \right] \tag{28}$$

where r is the distance between the source and the observation, θ is the elevation angle. $\eta = 376.7/\sqrt{\varepsilon_r}$ and $k = 2\pi\sqrt{\varepsilon_r}/\lambda$ are the homogeneous medium impedance and the wavenumber, respectively. The same technique is used for the evaluation of the magnetic field due to the excitation. This allows for limiting the integration versus k_ρ to about $20k_0$, where $k_0 = 2\pi/\lambda$ is the free-space wavenumber.
